# Impacts of Digital Care Programs for Musculoskeletal Conditions on Depression and Work Productivity: Longitudinal Cohort Study

**DOI:** 10.2196/38942

**Published:** 2022-07-25

**Authors:** Fabíola Costa, Dora Janela, Maria Molinos, Robert Moulder, Vírgilio Bento, Jorge Lains, Justin Scheer, Vijay Yanamadala, Steven Cohen, Fernando Dias Correia

**Affiliations:** 1 SWORD Health Inc Draper, UT United States; 2 Institute for Cognitive Science University of Colorado Boulder, CO United States; 3 Rovisco Pais Medical and Rehabilitation Centre Tocha Portugal; 4 Faculty of Medicine Coimbra University Coimbra Portugal; 5 Department of Neurological Surgery University of California San Francisco, CA United States; 6 Department of Surgery Frank H Netter School of Medicine Quinnipiac University Hamden, CT United States; 7 Department of Neurosurgery Hartford Healthcare Medical Group Westport, CT United States; 8 Department of Anesthesiology and Critical Care Medicine Johns Hopkins School of Medicine Baltimore, MD United States; 9 Department of Physical Medicine and Rehabilitation Uniformed Services University of the Health Sciences Bethesda, MD United States; 10 Neurology Department Centro Hospitalar e Universitário do Porto Porto Portugal

**Keywords:** musculoskeletal, pain, depression, anxiety, mental health, comorbidity, productivity, digital health, remote care, rehabilitation, telehealth, telemedicine, eHealth, digital health, digital care, multimodal, digital intervention, recovery, engagement, activities of daily living, work, job, occupational health, longitudinal cohort

## Abstract

**Background:**

Comorbidity between musculoskeletal (MSK) pain and depression is highly common, and is associated with a greater symptom burden and greater loss of work productivity than either condition alone. Multimodal care programs tackling both physical and mental health components may maximize productivity recovery and return to work. Digital delivery of such programs can facilitate access, ensure continuity of care, and enhance patient engagement.

**Objective:**

The aim of this study was to assess the impact of a completely remote multimodal digital care program (DCP) for MSK pain on mental health and work-related outcomes stratified by baseline depression levels.

**Methods:**

Ad hoc analysis of an interventional, single-arm, cohort study of individuals with MSK pain undergoing a DCP was performed. Three subgroups with different baseline depression severity levels were established based on responses to the Patient Health Questionnaire (PHQ-9): cluster 1 (score<5: minimal depression), cluster 2 (scores 5-10: mild depression), and cluster 3 (score≥10: moderate depression). The mean changes in depression, anxiety, fear-avoidance beliefs, work productivity, and activity impairment and adherence between baseline and end of program (8-12 weeks) were assessed across subgroups by latent growth curve analysis.

**Results:**

From a total of 7785 eligible participants, 6137 (78.83%) were included in cluster 1, 1158 (14.87%) in cluster 2, and 490 (6.29%) in cluster 3. Significant improvements in depression and anxiety scores were observed in clusters 2 and 3 but not in cluster 1, with average end-of-the program scores in clusters 2 and 3 below the initially defined cluster thresholds (score of 5 and 10, respectively). All clusters reported significant improvements in productivity impairment scores (mean changes from –16.82, 95% CI –20.32 to –13.42 in cluster 1 to –20.10, 95% CI –32.64 to –7.57 in cluster 3). Higher adherence was associated with higher improvements in depression in clusters 2 and 3, and with greater recovery in activities of daily living in cluster 3. Overall patient satisfaction was 8.59/10.0 (SD 1.74).

**Conclusions:**

A multimodal DCP was able to promote improvements in productivity impairment scores comparable to those previously reported in the literature, even in participants with comorbid depression and anxiety. These results reinforce the need to follow a biopsychosocial framework to optimize outcomes in patients with MSK pain.

**Trial Registration:**

ClinicalTrials.gov NCT04092946; https://clinicaltrials.gov/ct2/show/NCT04092946

## Introduction

Musculoskeletal (MSK) pain is highly prevalent worldwide, affecting hundreds of millions of individuals with disability and personal suffering, while imposing a great socioeconomic burden [1]. Comorbidity between MSK pain and depression is very common [2] due to shared pathophysiological mechanisms, which establishes a strong and complex bidirectional relationship [3,4]. Comorbid depression symptoms have been associated with an increased MSK pain symptom burden and impaired recovery of both conditions [5,6]. This negative synergistic effect translates into poor work productivity [7,8], either by impaired performance at work (presenteeism) or work absence (absenteeism); decreased general quality of life; medical complications; and subsequent additional care [7-9]. Comorbid depression and MSK pain are associated with higher health care expenditures [10] estimated at US $13,000 annually in the United States, which is almost double that estimated for chronic pain alone [11]. In the United States, the annual cost associated with productivity loss from depression amounts to US $1150 per individual [12] and US $44 billion [13] for society, while the indirect costs associated with MSK pain are estimated at US $264 billion [14].

Exercise-based physical therapy is a first-choice intervention to address MSK pain [15-17]. Current guidelines advise addressing depression (as well as other cognitive and psychological factors) as part of MSK pain management [15,16,18] through a biopsychosocial approach [19,20], including pain education, psychoeducation, or even specifically cognitive behavioral therapy (CBT). This biopsychosocial approach has been increasingly applied and is naturally evolving with the optimization and digitalization of health care. With more than 62.5% of the global population now able to access the internet (according to Worldwide Digital Population estimates as of January 2022 [21]), digital interventions may offer highly scalable solutions to deliver evidence-based interdisciplinary interventions [22], thereby democratizing access and improving the continuity of care in cases where specially trained health care practitioners may not be readily available [23], and also promoting adherence to treatment by facilitating therapeutic alliance (defined as collaboration between therapeutic participants to foster healing) [24,25]. Digital interventions have therefore been explored for the treatment of depression and MSK diseases [26,27]. In 2021, the US Department of Health and Human Services’ Substance Abuse and Mental Health Services Administration released an evidence-based resource guide system recommending the use of telehealth for people with serious mental health disorders such as depression, noting that the benefits of telehealth services in this context may extend beyond improvement in morbid psychological conditions, including chronic pain and pain-related disability [28].

To date, digital intervention studies have been focused on either pain and disability [29-31] or on mental health and pain [26,27,32,33], with only a few studies assessing the impact of either dimension (MSK pain and depression) on work-related productivity [27,34-36].

Previously, we reported a multimodal digital care program (DCP) that integrates physical therapy exercise-based management with a psychoeducational component, including CBT, that aims to encourage patients to develop self-management skills and strategies for their pain. This DCP has been validated in different MSK conditions in chronic [37], acute [38,39], and postsurgical contexts [40-43]. Herein, we aimed to assess mental health and work-related outcomes after a completely remote multimodal DCP for patients with MSK pain stratified by baseline depression levels.

We hypothesized that this multimodal DCP would be able to contribute to mental health and promote productivity impairment improvements, despite differences in the initial mental status of participants.

## Methods

### Study Design

This is an ad hoc analysis of a decentralized, single-arm ongoing study, focused on assessing clinical and engagement-related outcomes in patients with MSK pain after a home-based multimodal DCP.

### Ethics Approval

This study was prospectively approved by the New England Institutional Review Board (number 120190313) and was registered on ClinicalTrials.gov (NCT04092946) on September 17, 2019.

### Participants

Beneficiaries of employer health plans, older than 18 years of age, and suffering from MSK pain (either in the spine or in the upper or lower limbs) were invited to apply for SWORD Health’s DCP through a dedicated website (which preselected candidates with ability to interact with technologies). Exclusion criteria were: (1) a health condition (eg, cardiac, respiratory) incompatible with at least 20 minutes of light to moderate exercise; (2) receiving active treatment for cancer; (3) reporting new-onset, rapidly progressive loss of strength and/or numbness in the arms/legs; or (4) reporting an unexplained change in bowel or urinary function in the previous 2 weeks. Informed consent was obtained from all participants.

### Intervention

The DCP was delivered between August 1, 2020, and October 12, 2021. This completely remote DCP integrates individually tailored exercises and a psychoeducational component, which includes both education and CBT. Upon enrollment, each participant was assigned to a physical therapist who was responsible for program customization and asynchronous monitoring of patient performance. The exercise sessions consist of gradual progressive movement exposure and are performed through a Food and Drug Administration–listed class II medical device, including a tablet with a preinstalled app and wearable motion-tracking sensors. The tablet displays the prescribed exercises through audio/videos, while sensors digitize motion, providing real-time biofeedback along with instructions to guide patients during their sessions. Data obtained from the exercise sessions are stored on a cloud-based platform, being asynchronously monitored through a web-based portal by the assigned physical therapist who adjusts the exercises according to the patient’s progression. Participants were recommended to perform 3 exercise sessions per week, with an expected program duration ranging between 8 and 12 weeks depending on the condition (although early discharge was possible depending on physical therapist assessment). Absence of an exercise session for 28 consecutive days resulted in classification of the participant as a dropout. Participants were still considered if they were compliant with the intervention but failed to complete a given reassessment survey.

The psychoeducational component was developed under current clinical guidelines and research [17,44,45]. Educational articles were delivered through the app, covering a broad range of MSK pain–related topics, explaining pain and pain management. The CBT program consisted of self-guided interactive modules delivered through the smartphone app. This program was created by a multidisciplinary team including psychiatrists and psychologists based on third-generation CBT techniques, including mindfulness, acceptance and commitment therapy, and empathy-focused therapy. The CBT program was specifically designed to address fear avoidance, pain reconceptualization, active coping skills, as well as anxiety and depression associated with MSK pain. Bidirectional communication between participants and physical therapists, after exercise sessions or on demand, were ensured through a built-in secure chat feature on the smartphone app, and through synchronous video calls between the physical therapist and participant to facilitate therapeutic alliance, adjust treatment, and monitor potential adverse events.

### Outcome Metrics

Self-reported assessments were collected at baseline, 4, 8, and 12 weeks, while mean changes were calculated between baseline and program end. Outcomes included mean change of (1) depression, measured by the 9-item Patient Health Questionnaire (PHQ-9; range 0-27) [46,47]; (2) anxiety, measured by the 7-item Generalized Anxiety Disorder (GAD-7) questionnaire (range 0-21) [48]; (3) Fear-Avoidance Beliefs Questionnaire for Physical Activity (FABQ-PA), comprising a total of 5 items each with a 7-option Likert scale (range 0-24) [49]; (4) Work Productivity and Activity Impairment (WPAI) questionnaire (0-100), including WPAI-overall (presenteeism and absenteeism from work), WPAI-work (presenteeism), WPAI-time (absenteeism, evaluated in employed participants), and WPAI-activity (for nonwork-related activity impairment in all participants) [50]; and (5) adherence, assessed through the number of completed sessions per week, total exercise time (minutes), communication frequency with the physical therapist, and overall satisfaction (points) through the question “On a scale from 0 to 10, how likely is it that you would recommend this intervention to a friend or neighbor? (from 0, not at all likely to 10, extremely likely). Overall, higher self-reported outcomes scores indicate higher severity.

### Safety and Adverse Events

Participants were asked to grade the severity of pain and fatigue (from 0 to 10, with 10 being the most severe) on all exercise sessions to allow monitoring by the physical therapists. All communication channels were available for participants to report any adverse events. PHQ-9 and GAD-7 scores were used not only to guide the intervention approach but also to direct members to psychological and/or psychiatric care when needed, following the US Department of Health and Human Services guidelines.

### Statistical Analysis

Study population demographics and clinical data, as well as usability metrics (number of sessions per week, total exercising time) were characterized through descriptive statistics. Differences in baseline characteristics between clusters (see below) were assessed through *χ*^2^ tests for categorical variables and independent-samples *t* tests or one-way analysis of variance with Bonferroni posthoc correction for continuous variables.

Considering that depression has been reported as an important prognosis factor [51,52], PHQ-9 was used as a clustering variable, applying the thresholds <5 for no depression symptoms, 5-10 for mild symptoms, and ≥10 for moderate/severe symptoms, according to Kroenke et al [53].

For longitudinal data analysis, the latent growth curve was applied, which is a methodology in the same family as linear mixed-effects modeling, with the advantages of providing a measure of model fitness (eg, how well the model explains the data set) [54], and allowing the use of full information maximum likelihood (FIML) to address missing data. FIML has been shown to outperform other modern imputation models such as multiple imputation by chained equations (MICE) and listwise deletion [54-56]. FIML estimation considers all available data in each time point from all participants [55,56].

Latent growth curve analysis uses a structural equation model [57] to estimate the trajectories of outcomes over time based on individual trajectories and considering time as a continuous variable. This provides an estimate of the average trajectory (and respective pace of change) and individual variation around that trajectory over time (see Figure S1 in Multimedia Appendix 1).

The analysis was performed as an intent-to-treat analysis both for unfiltered cases and filtering for WPAI>0 points at baseline. Impact of training time on outcomes was modeled using cumulative training time as a time-invariant covariate. A conditional analysis was also performed to assess the influence of age, sex, and BMI as covariates. Models were adjusted for these covariates, which were fit as random effects allowing each to vary between individuals. All models were estimated with a robust sandwich estimator for standard errors.

Logistic regression was used to assess the relationship between baseline depression and productivity changes.

Significance levels were set at *P*<.05 in all analyses. Latent growth curve analysis was coded using R (version 1.4.1717) and all other analyses were performed using SPSS (version 17.0).

## Results

### Participant Characteristics

**Figure 1:**
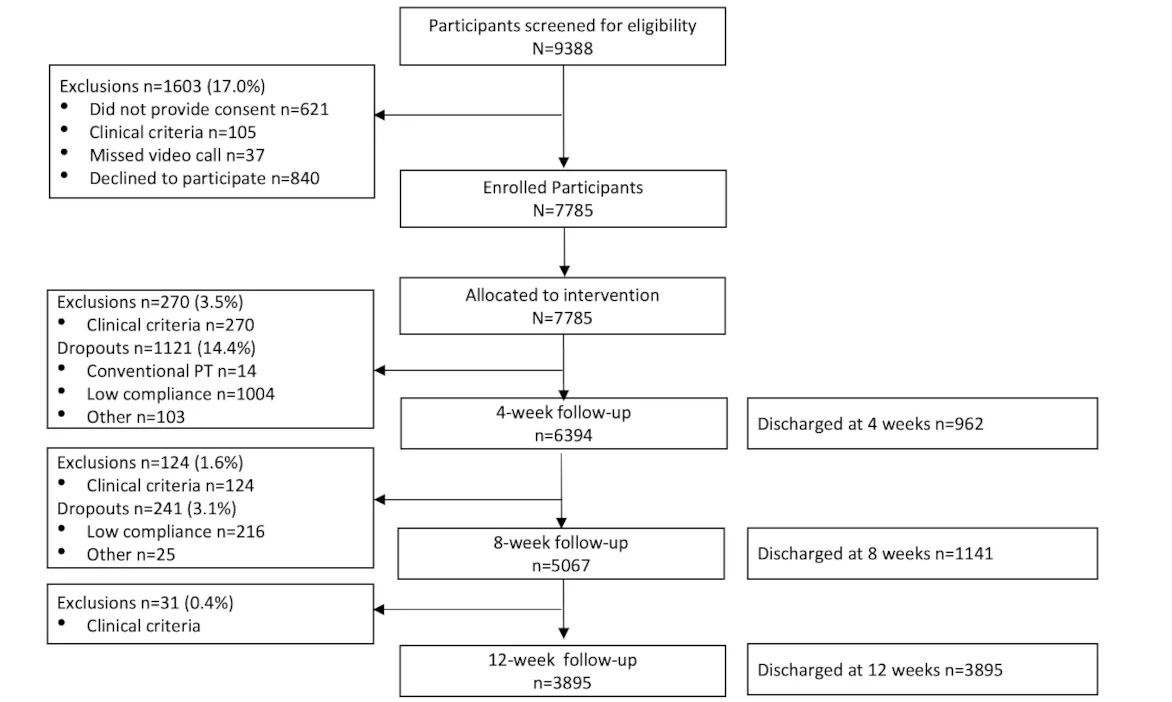
Flowchart of the study.

In total, 9388 participants were screened for eligibility, 621 (6.61%) of whom did not provide consent for research and 982 (10.46%) of whom were excluded (105 for clinical criteria, 37 participants missed their video call, and 840 declined to participate in the program during the video call) (Figure 1). In total, 7785 participants from all 50 states within the United States started the program. Overall, the completion rate was 77.05% (5998/7785), with 16.04% (962/5998), 19.02% (1141/5998), and 64.94% (3895/5998) participants discharged at 4, 8, and 12 weeks, respectively.

Using PHQ-9 as a clustering variable, 3 clusters were created: <5, 5-10, and ≥10 points, according to Kroenke et al [53]. Baseline demographics of each cluster are reported in Table 1 and those of the entire cohort are provided in Table S1 of Multimedia Appendix 2.

**Table 1:** Baseline characteristics of study participants in each depression-related cluster.

Characteristic		Cluster 1, PHQ-9^a^ score <5 (n=6137)	Cluster 2, PHQ-9 score 5-10 (n=1158)	Cluster 3, PHQ-9 score ≥10 (n=490)	*P* value	
Age (years), mean (SD)		51.4 (12.7)	50.0 (13.4)	48.7 (13.4)	<.001	
**Age category (years), n (%)**						<.001
	<25	43 (0.7)	16 (1.4)	8 (1.6)		
	25-40	1339 (21.8)	298 (25.7)	146 (29.8)		
	40-60	3172 (51.7)	561 (48.4)	225 (45.9)		
	>60	1583 (25.8)	283 (24.4)	111 (22.7)		
**Gender, n (%)**						<.001
	Woman	3276 (53.4)	702 (60.6)	297 (60.6)		
	Man	2848 (46.4)	453 (39.1)	191 (39.0)		
	Nonbinary	13 (0.2)	3 (0.3)	2 (0.4)		
BMI^b^, mean (SD)		28.8 (6.3)	30.6 (7.1)	32.9 (7.9)	<.001	
**BMI category^b^, n (%)**						<.001
	Underweight (<18.5)	46 (0.8)	10 (0.9)	3 (0.6)		
	Normal (18.5-25)	1789 (29.2)	247 (21.4)	88 (18.0)		
	Overweight (25-29)	2145 (35.0)	362 (31.3)	122 (25.0)		
	Obese (30-39)	1775 (29.0)	412 (35.7)	197 (40.4)		
	Obese grade III (>40)	367 (6.0)	124 (10.7)	78 (16.0)		
**Conditions addressed, n (%)**						<.001
	Spine	2961 (48.2)	626 (54.1)	314 (64.1)		
	Lower limb	1739 (28.3)	310 (26.8)	108 (22.0)		
	Upper limb	1437 (23.4)	222 (19.2)	68 (13.9)		
**Pain duration^c^, n (%)**						<.001
	Acute (<12 weeks)	1620 (26.5)	235 (20.4)	68 (13.9)		
	Chronic (>12 weeks)	4492 (73.5)	919 (79.6)	422 (86.1)		
**Employment status, n (%)**						<.001
	Employed (part-time or full-time)	5260 (85.7)	992 (85.7)	364 (74.3)		
	Unemployed	877 (14.3)	166 (14.3)	126 (25.7)		
**Outcome measures, mean (SD)**						
	Pain level	4.67 (1.98)	5.10 (1.95)	5.73 (1.97)	<.001	
	Analgesics, n (%)	1861 (30.3)	464 (40.1)	212 (43.3)	<.001	
	Surgery intent	10.26 (19.52)	11.97 (21.41)	17.32 (26.86)	<.001	
	FABQ-PA^d,e^	10.27 (5.99)	11.63 (5.82)	12.72 (5.96)	<.001	
	GAD-7^f^	1.82 (2.88)	6.17 (4.23)	11.66 (5.62)	<.001	
	PHQ-9	0.86 (1.25)	6.93 (1.63)	14.84 (3.74)	<.001	
	WPAI^g^-overall^h^	14.78 (20.48)	23.94 (23.60)	40.87 (30.51)	<.001	
	WPAI-work^h^	13.86 (19.17)	22.08 (22.09)	38.04 (28.62)	<.001	
	WPAI-time^h^	1.98 (10.52)	4.36 (15.29)	12.02 (26.16)	<.001	
	WPAI-activity	25.43 (23.97)	34.47 (25.04)	50.49 (26.29)	<.001	

^a^PHQ-9: 9-item Patient Health Questionnaire.

^b^n=20 missing values.

^c^n=29 missing values.

^d^FABQ-PA: Fear-Avoidance Beliefs Questionnaire for Physical Activity.

^e^n=10 missing values.

^f^GAD-7: 7-item Generalized Anxiety Disorder questionnaire.

^g^WPAI: Work Productivity and Activity Impairment questionnaire.

^h^N=6030.

Participants were unevenly distributed across the three clusters, with 6137, 1158, and 490 participants in cluster 1, 2, and 3, respectively. Several demographic characteristics were significantly different between clusters, namely the proportion of women, unemployed, younger participants, acuity, or those with higher BMI, which were progressively more frequent with increasing levels of depression (from cluster 1 to 2 and to 3, *P*<.001; Table 1). Significant baseline differences were also observed in clinical characteristics between clusters, as cluster 1 had no impairment in mental health (GAD-7 scores below 2 and FABQ 10.3, SD 6.0), and low levels of productivity impairment (WPAI-overall ~15%, SD 20.5 impairment). Cluster 2 reported mild anxiety and fear-avoidance beliefs (GAD-7 above 5 and FABQ 11.3, SD 5.8) and work difficulties (WPAI overall ~22%, SD 24.2 impairment), while cluster 3 presented with the highest anxiety (GAD-7 above 10) and greatest fear-avoidance beliefs (FABQ 12.7, SD 6.0) measures, along with greater impairments in productivity (40.87%, SD 30.5) or activities of daily living (50.5%, SD 26.3; *P*<.001; Table 1).

### Clinical Outcomes

#### Overview

Changes in clinical outcomes over time are presented in Table 2, the model is presented in Table S2 of Multimedia Appendix 3, and the impact of covariates in the model is reported in Table S3 of Multimedia Appendix 4.

**Table 2:** Changes in clinical outcomes between baseline and end of program: intent-to-treat analysis.

Variable	Cluster 1				Cluster 2				Cluster 3		
	Baseline, mean (95% CI)	Change, mean (95% CI)	*P* value	Baseline, mean (95% CI)		Change, mean (95% CI)	*P* value	Baseline, mean (95% CI)		Change, mean (95% CI)	*P* value
PHQ-9^a^	0.81 (0.77 to 0.86)	–0.03 (–0.25 to 0.18)	.77	6.58 (6.44 to 6.71)		–3.36 (–4.26 to 2.45)	<.001	13.98 (13.46 to 14.5)		–4.09 (–6.87 to –1.32)	.004
GAD-7^b^	1.44 (1.35 to 1.53)	–0.43 (–0.68 to –0.18)	<.001	5.44 (5.07 to 5.8)		–2.25 (–3.14 to –1.36)	<.001	10.73 (9.99 to 11.47)		–2.24 (–4.49 to 0.01)	.05
FABQ-PA^c^	10.4 (10.18 to 10.62)	–2.63 (–3.17 to –2.09)	<.001	11.49 (10.98 to 12.01)		–2.33 (–3.62 to –1.04)	<.001	12.67 (11.92 to 13.42)		–0.7 (–2.84 to 1.44)	.52
WPAI^d^-overall	13.9 (13.11 to 14.7)	–6.84 (–8.82 to –4.86)	<.001	21.87 (19.39 to 24.35)		–11.33 (–16.6 to –6.06)	<.001	39.68 (34.86 to 44.5)		–12.34 (–23.65 to –1.03)	.03
WPAI-overall^e^	27.26 (26.1 to 28.41)	–16.82 (–20.23 to –13.42)	<.001	31.00 (28.26 to 33.74)		–19.11 (–25.75 to –12.47)	<.001	45.43 (40.62 to 50.24)		–20.1 (–32.64 to –7.57)	.002
WPAI-activity	22.57 (21.74 to 23.4)	–10.08 (–11.95 to –8.21)	<.001	30.99 (28.74 to 33.23)		–13.14 (–18.22 to –8.06)	<.001	46.35 (42.88 to 49.82)		–5.07 (–14.08 to 3.95)	.27
WPAI-activity^e^	32.04 (31.14 to 32.95)	–15.66 (–17.98 to –13.34)	<.001	36.28 (34.08 to 38.48)		–16.17 (–21.57 to –10.77)	<.001	49.5 (46.16 to 52.83)		–7.01 (–16.52 to 2.5)	.15
WPAI-work	13.04 (12.3 to 13.79)	–6.20 (–8.00 to –4.39)	<.001	20.78 (18.4 to 23.16)		–11.37 (–16.17 to –6.57)	<.001	37.13 (32.59 to 41.67)		–13.54 (–24.42 to –2.65)	.01
WPAI-work^e^	26.06 (24.98 to 27.13)	–15.24 (–18.35 to –12.12)	<.001	29.58 (26.93 to 32.24)		–19.36 (–25.29 to –13.42)	<.001	42.97 (38.44 to 47.51)		–21.9 (–34.43 to –9.37)	<.001
WPAI-time	2.02 (1.58 to 2.46)	–0.98 (–2.09 to 0.12)	.06	3.67 (2.01 to 5.34)		–1.42 (–3.93 to 1.09)	.23	13.45 (8.94 to 17.97)		–5.63 (–11.05 to –0.21)	.03
WPAI-time^e^	27.02 (22.52 to 31.52)	–28.54 (–39.11 to –17.96)	<.001	25.65 (17.24 to 34.06)		–20.5 (–31.84 to –9.16)	<.001	46.1 (35.5 to 56.7)		–23.13 (–43.29 to –2.98)	.02

^a^PHQ-9: 9-item Patient Health Questionnaire.

^b^GAD-7: 7-item Generalized Anxiety Disorder questionnaire.

^c^FABQ-PA: Fear-Avoidance Beliefs Questionnaire for Physical Activity.

^d^WPAI: Work Productivity and Activity Impairment questionnaire.

^e^Filtered for scores>0 at baseline.

#### Depression

Significant improvement was observed on the PHQ-9 for clusters 2 and 3, but not for cluster 1, which reported minimal depression symptoms at baseline (Table 2). Average end-of-the program scores for depression in clusters 2 and 3 decreased to levels below the initially defined lower cluster thresholds (5 and 10 for clusters 2 and 3, respectively; end scores: 3.22, 95% CI 2.31-4.13 and 9.89, 95% CI 7.15-12.62, respectively).

Significant differences in PHQ-9 score changes were observed between all clusters (*P*<.001, Table 3). Women reported higher levels of depression than men in cluster 1 (*P*<.001), but recovered at a faster pace in clusters 1 and 2. Older participants in cluster 3 recovered from depression symptoms at a slower pace (*P*=.04).

**Table 3:** Differences in clinical outcomes change stratified by baseline depression level.

Outcome	Cluster 1 versus cluster 2		Cluster 1 versus cluster 3		Cluster 2 versus cluster 3	
	Difference (95% CI)	*P* value	Difference (95% CI)	*P* value	Difference (95% CI)	*P* value
PHQ-9^a^	–2.44 (–3.37 to –1.51)	<.001	–9.11 (–11.85 to –6.36)	<.001	–6.66 (–9.55 to –3.78)	<.001
GAD-7^b^	–2.17 (–3.08 to –1.25)	<.001	–7.47 (–9.71 to –5.23)	<.001	–5.3 (–7.69 to –2.92)	<.001
FABQ-PA^c^	–1.39 (–2.79 to 0.01)	.05	–4.2 (–6.43 to –1.96)	<.001	–2.81 (–5.33 to –0.28)	.03
WPAI^d^-overall	–3.48 (–8.96 to 2.01)	.22	–20.28 (–31.72 to –8.84)	<.001	–16.8 (–29.21 to –4.4)	.008
WPAI-overall^e^	–2.29 (–5.17 to 9.75)	.55	–3.28 (–9.71 to –16.28)	.62	–1.00 (–13.19 to 15.18)	.07
WPAI-activity	–5.35 (–10.74 to 0.04)	.05	–28.79 (–37.88 to –19.7)	<.001	–23.43 (–33.69 to –13.18)	<.001
WPAI-activity^e^	–0.51 (–5.37 to 6.39)	.87	–8.65 (–18.44 to 1.14)	.08	–9.16 (–20.10 to 1.77)	.11
WPAI-work	–2.57 (–7.53 to 2.38)	.31	–16.75 (–27.85 to –5.64)	.003	–14.18 (–26.08 to –2.27)	.02
WPAI-work^e^	4.12 (–2.58 to 10.83)	.23	6.66 (–6.25 to 19.57)	.31	2.54 (–11.33 to 16.40)	.72
WPAI-time	–1.21 (–3.75 to 1.32)	.35	–6.78 (–12.53 to –1.03)	.02	–5.57 (–11.69 to 0.55)	.08
WPAI-time^e^	–8.04 (–23.54 to 7.47)	.31	–5.41 (–28.17 to 17.36)	.64	2.63 (–20.50 to 25.76)	.84

^a^PHQ-9: 9-item Patient Health Questionnaire.

^b^GAD-7: 7-item Generalized Anxiety Disorder questionnaire.

^c^FABQ-PA: Fear-Avoidance Beliefs Questionnaire for Physical Activity.

^d^WPAI: Work Productivity and Activity Impairment questionnaire.

^e^Filtered for scores>0 at baseline.

#### Anxiety

Cluster 1 did not report significant anxiety levels at baseline; thus, the observed change was not meaningful. Clusters 2 and 3 showed statistically significant improvements after the DCP (Table 3), ending the program with lower levels of anxiety than at baseline (end scores: 3.18, 95% CI 2.31-4.06 and 8.49, 95% CI 6.27-10.71, respectively).

Changes in anxiety levels were again significantly different among clusters (*P*<.001, Table 3). Women reported greater anxiety levels than men at baseline in clusters 1 and 2, whereas a faster-paced recovery was observed across all clusters for women (*P*<.001, Table S3 in Multimedia Appendix 4). Older participants in cluster 3 recovered from anxiety at a slower pace (*P*=.02). BMI did not influence mental health improvement trajectories in any cluster (Table S3 in Multimedia Appendix 4).

#### Fear-Avoidance Beliefs

Statistically significant improvements were observed in clusters 1 and 2, with a mean change of approximately –2.50 in both clusters (*P*<.001, Table 2). No significant improvement was observed in cluster 3 (*P*=.52, Table 2). BMI did not influence FABQ improvement trajectories in any cluster (Table S3 in Multimedia Appendix 4).

#### Work Productivity

Baseline impairments in work and in activities of daily living were progressively higher from cluster 1 to cluster 3 (Table 1). Across all WPAI scores, similar mean changes were observed between clusters (Table 3) when filtering for participants reporting impairments at baseline, despite different baseline values (Table 2).

Age and BMI did not consistently influence productivity impairment improvement across all clusters (Table S3 in Multimedia Appendix 4). Among women, we observed deacceleration in the improvement pace toward the end of the program for the WPAI overall score (Table S3 in Multimedia Appendix 4) as well as higher baseline scores paired with a slower recovery pace in WPAI activity.

Productivity impairment (WPAI-overall score) improved significantly across all clusters with mean changes ranging from –16.82 (95% CI –20.32 to –13.42) in cluster 1 and –19.11 (95% CI –25.75 to –12.47) in cluster 2 to –20.10 (95% CI –32.64 to –7.57) in cluster 3 when filtering for participants with reported impairment at baseline (WPAI>0). A significant, albeit small, correlation between baseline PHQ-9 values and WPAI change was only observed in cluster 3 (0.30, *P*=.01).

### Adherence and Usability-Related Outcomes

The average number of sessions per week was 2.7 (SD 1.39) across all clusters, but with individuals in clusters 2 and 3 showing slightly lower adherence (average of 2.6 sessions per week, SD 1.3; *P*<.001). This paralleled differences in the amount of time dedicated to exercise between clusters, which ranged from 552.4 minutes in cluster 1 to 384.7 minutes in cluster 3 (Table 4, *P*<.001). The influence of training time on outcome changes was estimated regarding trajectory slopes (Table 5).

In cluster 1, training time was significantly associated with FABQ reduction (*P*<.001) and improvement of WPAI-activity (*P*=.001). In cluster 2, increased training time was significantly associated with improvements in depression (*P*=.03) and FABQ (*P*=.002), but with no significant effect on productivity. In cluster 3, increased training times were significantly associated with greater improvements in mental health (depression [PHQ-9, *P*=.005], anxiety [GAD-7, *P*=.001], fear avoidance [FABQ, *P*=.003]) and in activities of daily living impairment (WPAI-activity, *P*<.001).

Regarding communication channels, the app chat was the preferred mode of contact, with an average of 9.1 days with contact. There were no significant differences between clusters (*P*=.21, Table 4), with cluster 3 showing a higher number of exchanged messages (Table 4). On average, 1.7 (SD 2.7) calls were made, which did not significantly differ between clusters (*P*=.22). Each participant on average engaged with 4.3 (SD 7.0) pieces of educational and CBT content, with no significant difference observed between clusters. Overall, the average satisfaction score was 8.6 (SD 1.7), which again did not significantly differ among the three clusters.

**Table 4:** Adherence outcome measures of the entire cohort and particular clusters.

Usability outcomes	Entire cohort, mean (SD)	Cluster 1, mean (SD)	Cluster 2, mean (SD)	Cluster 3, mean (SD)	*P* value
Number of sessions per week	2.7 (1.39)	2.7 (1.40)	2.6 (1.31)	2.6 (1.32)	<.001
Total exercising time, minutes	531.0 (522.3)	552.4 (537.4)	479.1 (473.6)	384.7 (392.6)	<.001
Average satisfaction (NRS^a^, 0-10)	8.6 (1.74)^b^	8.6 (1.72)	8.5 (1.77)	8.6 (1.98)	.59
Number of days with contact (chat)	9.1 (11.6)	9.1 (11.6)	8.8 (11.5)	10.0 (11.7)	.21
Number of messages exchanged	23.74 (27.10)	23.30 (26.86)	24.37 (27.20)	28.37 (29.65)	*.*02
Number of educational articles per week	4.29 (7.0)	4.24 (7.0)	4.56 (6.9)	4.32 (7.2)	.37

^a^NRS: numerical rating scale.

**Table 5:** Effect of cumulative training time on the slopes of recovery trajectories for the different outcome variables.^a^

Variable	Cluster 1			Cluster 2			Cluster 3	
	Estimate	*P* value	Estimate		*P* value	Estimate		*P* value
PHQ-9^b^	–0.01	.18	–0.06		.03	–0.24		.005
GAD-7^c^	–0.01	.23	–0.05		.13	–0.20		.001
FABQ-PA^d^	–0.07	<.001	–0.13		.002	–0.21		.003
WPAI^e^-overall^f^	0.01	.92	0.13		.57	–0.56		.19
WPAI-activity^f^	–0.24	.001	–0.30		.10	–1.31		<.001
WPAI-work^f^	–0.03	.78	0.15		.48	–0.46		.29
WPAI-time^f^	0.39	.29	0.36		.34	–0.64		.49

^a^Negative values refer to more sharp slopes, indicating faster change over time.

^b^PHQ-9: 9-item Patient Health Questionnaire.

^c^GAD-7: 7-item Generalized Anxiety Disorder questionnaire.

^d^FABQ-PA: Fear-Avoidance Beliefs Questionnaire for Physical Activity.

^e^WPAI: Work Productivity and Activity Impairment questionnaire.

^f^Filtered for scores>0 at baseline.

## Discussion

### Principal Findings

Depression has been reported to be an important prognostic factor in MSK pain management [58,59]. By clustering patients according to baseline depression, we created three distinct groups, where other concomitant psychological factors (anxiety and fear-avoidance behaviors) with known prognostic value [5,60,61] were also observed at similar levels of severity. This is in accordance with the general population demographics, where depression is accompanied by other psychological/cognitive impairments [62,63], particularly in those with MSK pain [64]. Importantly, other demographic characteristics known to have prognostic value were distributed differently between clusters. Cluster 3 patients, besides suffering from more severe mental distress, were also more likely to be women [19], have a higher BMI [65], and be unemployed [66]. Cluster 2 contained these same factors at lower proportions but had a higher proportion of older participants [60], while cluster 1 tended to have more patients devoid of known risk factors.

Gender identification as a woman and older age were the predominant prognostic factors impacting both baseline and change in mental health status, while productivity was mainly influenced by gender identification as a woman. These results highlight the need to fine-tune programs to the specific needs of women, since this population is frequently identified as being more prone to psychological distress and prolonged MSK pain [67,68].

Notably, we observed that the improvements in mental health scores in clusters 2 and 3 resulted in average scores at the end of the program below the threshold used to define those clusters: cluster 2 from mild depression symptoms to minimal or no symptoms (<5) and cluster 3 from moderate to mild symptoms (<10). Overall, greater improvements were noted in cluster 3, demonstrating that multimodal DCP can not only impact physical health but also mental health in individuals with MSK pain and moderate mental health comorbidities.

### Comparison to Prior Work

The observed improvements in mental health scores were higher than those previously reported by other multimodal telerehabilitation interventions [69,70], and were in line with those previously reported by us [37,39] and others [27,32,34,71]. Similar to the present study, Wang et al [71] reported a significant decrease in anxiety symptoms, allowing the transition to a lower level of anxiety according to established thresholds after a telerehabilitation program combining exercise with coaching.

A correlation between baseline depression and WPAI change was only observed in cluster 3, which might suggest that the prognostic value of depression may be dependent on higher severity stages [72,73] or that it is more relevant in chronic conditions [8,72,74]. However, it may also reflect that there is little room for improvement in psychological indices in patients with little baseline psychopathology.

Regarding productivity, all clusters had significant WPAI improvements, independent of the recoveries reported for fear-avoidance beliefs assessment, which in this study appeared to be a poor predictor for work-related productivity recovery. In fact, while fear-avoidance belief has been systematically associated with disability and pain [75-77], its correlation with work-related outcomes might only be observed in those with high FABQ or FABQ-work subscale scores [78]. Similar improvements in productivity were reported by Bailey et al [34] in a cohort study involving more than 10,000 participants with MSK pain, either with or without depression symptoms at baseline. Overall, the results are supportive of the application of multimodal/biopsychosocial approaches to address psychological (mal)adaptation to the pain condition [61,79,80] and maximize treatment outcomes, as previously reported with other telerehabilitation interventions [81,82].

Interestingly, the improvement observed for productivity was not replicated to the same extent in activities of daily living, particularly in cluster 3. The observed difference might be explained by the high level of depression symptoms and also by the greater prevalence of participants who were women, as highlighted by the conditional analysis, with women still being more frequently responsible for family and household activities [83,84].

The importance of patient compliance to obtain clinically meaningful outcomes is well-established [85,86]. We observed high compliance with exercise sessions across clusters, even in cluster 3 where, besides higher depression levels, >50% of participants were obese, a known factor for reduced engagement [65,87]. Herein, increased amounts of time dedicated to exercise sessions were associated with greater improvements in mental health and activities of daily living recovery, as previously reported [88-90].

However, we observed lower adherence to the psychoeducational program than anticipated, despite high adherence with other components of the program, high satisfaction levels, and frequent communication with the assigned physical therapist. The same challenges have been highlighted by other authors [91], suggesting that additional innovation to stimulate engagement in such components might further contribute to better outcomes [92-94]. Communication is key for establishing a therapeutic alliance (defined as collaboration between therapeutic participants to facilitate healing), which in turn is key for improved outcomes [60,95-97]. Digital interventions have been reported to promote similar or even better therapeutic alliance than in-person interventions [25]. Herein, communication between physical therapists and individuals was frequent, highlighting the convenience of the app chat. Further studies are needed to clarify the extent to which this accessible and available communication system impacts outcome.

### Strengths and Limitations

Strengths of the study included the large sample size derived from a real-world context and the nature of the intervention: a multicomponent DCP managed by physical therapists combining exercises with real-time biofeedback within a biopsychosocial framework [17,95]. The digital format favors accessibility, while the regular communication with the same physical therapist may enhance adherence and maximize clinical outcomes [86,98]. The high adherence reported herein was objectively assessed to minimize social desirability response bias. Other strengths were the assessment of productivity through validated and widely used measures, and the inclusion of a heterogeneous cohort from geographically diverse states.

The major limitation refers to the study design that did not include a control group and thus does not allow us to determine the degree to which the various components of the program may have contributed to its overall reported changes, and whether all components benefit patients alike. Nevertheless, this study focused on an exploratory analysis of real-world data to support further research. Some variability was observed in terms of DCP participation and completion rates. However, the statistical methodologies chosen took into account the inclusion of real-world data, with missing data being handled through FIML, a method robust to attrition bias. This study included both emotional and cognitive outcomes; however, the inclusion of other psychosocial variables or tracking of mental-specific pharmacologic treatments could improve statistical models and further explain the variance, namely in the productivity measures.

### Conclusions

A multimodal DCP was able to promote significant improvements in productivity, comparable to those previously reported in the literature, even in participants with comorbid depression and anxiety at baseline. These results reinforce the need to follow a biopsychosocial framework to optimize outcomes in patients with MSK pain to maximize return to work.

## Appendix

Multimedia Appendix 1 Example path diagram for the latent growth curve (LGC) models used in the current study (Figure S1).

Multimedia Appendix 2 Table S1. Baseline characteristics of the entire cohort.

Multimedia Appendix 3 Table S2. Unconditional latent growth curve analysis: intent-to-treat analysis.

Multimedia Appendix 4 Table S3. Conditional growth-mixture modeling analysis: intent-to-treat analysis.

Multimedia Appendix 5 Overview of the exercise component framework, with illustrative exercise categories, progression principles, and dosage characteristics.

## Abbreviations

CBT: cognitive behavioral therapy
DCP: digital care program
FABQ-PA: Fear-Avoidance Beliefs Questionnaire for Physical Activity
FIML: full information maximum likelihood
GAD-7: Generalized Anxiety Disorder 7-item questionnaire
MICE: multiple imputation by chained equations
MSK: musculoskeletal
PHQ-9: Patient Health 9-item questionnaire
WPAI: Work Productivity and Activity Impairment questionnaire

## Data Availability

All relevant data are included in the article or available as supplementary material. The protocol, deidentified data, and analysis codes may be provided upon request to the corresponding author.
