# Addendum: Impacts of Digital Care Programs for Musculoskeletal Conditions on Depression and Work Productivity: Longitudinal Cohort Study

**DOI:** 10.2196/97493

**Published:** 2026-08-27

**Authors:** Fabíola Costa, Dora Janela, Maria Molinos, Robert Moulder, Virgílio Bento, Jorge Lains, Justin Scheer, Vijay Yanamadala, Steven Cohen, Fernando Dias Correia

**Affiliations:** 1 Sword Health Inc New York, NY United States; 2 Institute for Cognitive Science, University of Colorado Boulder, CO United States; 3 Rovisco Pais Medical and Rehabilitation Centre Tocha Portugal; 4 Faculty of Medicine, Coimbra University Coimbra Portugal; 5 Department of Neurological Surgery, University of California San Francisco, CA United States; 6 Department of Surgery, Frank H Netter School of Medicine, Quinnipiac University Hamden, CT United States; 7 Department of Neurosurgery, Hartford Healthcare Medical Group Westport, CT United States; 8 Department of Anesthesiology and Critical Care Medicine, Johns Hopkins School of Medicine Baltimore, MD United States; 9 Department of Physical Medicine and Rehabilitation, Uniformed Services University of the Health Sciences Bethesda, MD United States; 10 Neurology Department, Centro Hospitalar e Universitário do Porto Porto Portugal

In “Impacts of Digital Care Programs for Musculoskeletal Conditions on Depression and Work Productivity: Longitudinal Cohort Study” [1], additional clarification has been added regarding the exercise prescription to enhance transparency and clarity of the intervention provided.

To complement the description in the original manuscript, Multimedia Appendix 5 has been added (included here as Multimedia Appendix 1) to provide an overview of the exercise interventions commonly delivered within the digital care program, including representative exercise categories, progression principles, and illustrative dosage parameters. Detailed descriptions of condition-specific implementations of digital care programs have been reported in previously published studies [2,3], cited in the Introduction section of the original article.

These additions do not affect the study design, analyses, or conclusions of the original publication.

## Appendix

Multimedia Appendix 1 Overview of the exercise component framework, with illustrative exercise categories, progression principles, and dosage characteristics.

## References

[1] Costa Fabíola, Janela Dora, Molinos Maria, Moulder Robert, Bento Vírgilio, Lains Jorge, Scheer Justin, Yanamadala Vijay, Cohen Steven, Dias Correia Fernando (2022). mpacts of digital care programs for musculoskeletal conditions on depression and work productivity: longitudinal cohort study. J Med Internet Res.

[2] Correia Fernando Dias, Nogueira André, Magalhães Ivo, Guimarães Joana, Moreira Maria, Barradas Isabel, Teixeira Laetitia, Tulha José, Seabra Rosmaninho, Lains Jorge, Bento Virgilio (2018). Home-based rehabilitation with a novel digital biofeedback system versus conventional in-person rehabilitation after total knee replacement: a feasibility study. Sci Rep.

[3] Correia Fernando Dias, Molinos Maria, Luís Sara, Carvalho Diana, Carvalho Carlos, Costa Pedro, Seabra Rosmaninho, Francisco Gerard, Bento Virgílio, Lains Jorge (2022). Digitally assisted versus conventional home-based rehabilitation after arthroscopic rotator cuff repair: a randomized controlled trial. Am J Phys Med Rehabil.

