# Supplementary material for: Impacts of Digital Care Programs for Musculoskeletal Conditions on Depression and Work Productivity: Longitudinal Cohort Study
Source: J Med Internet Res. 2022 Jul 25;24(7):e38942. doi: 10.2196/38942 (PMC9361146; doi:10.2196/38942)
Supplement: Multimedia Appendix 1 [file jmir_v24i7e38942_app1.docx]

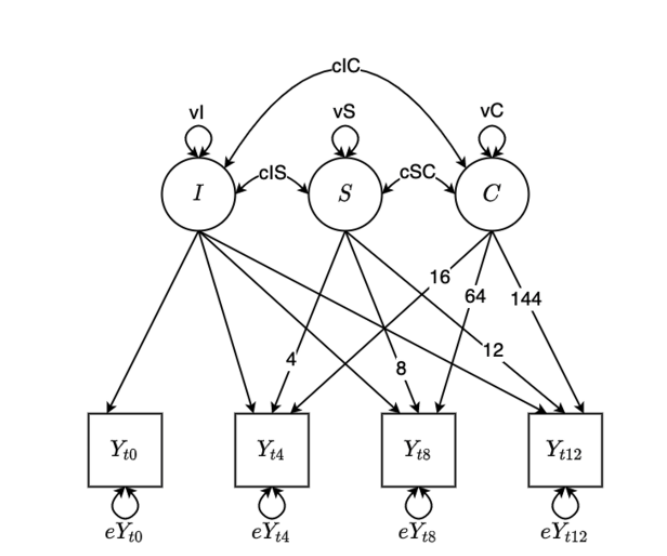


Figure S1. Example path diagram for the latent growth curve (LGC) models used in the current study.

LGCs are a form of structural equation model for modeling longitudinal processes. Squares

represent outcome variables at baseline, 4 weeks, 8 weeks, and 12 weeks. Circles represent

average latent intercept (I), slope (S), and curvature (C) components across all participants.

Single-headed arrows represent fixed loadings relating the outcome variables to the latent

components. Double-headed arrows represent either variance, covariance, or error variance

parameters: variance of the intercept (vI), slope (vS) and curvature (vC); covariance between

intercept and slope (cIS), slope and curvature (cSC), and intercept and curvature (cIC); error

variance across measurement time points - at baseline (eYt0), 4 (eYt4), 8 (eYt8) and 12 (eYt12)

weeks.

Latent growth curve (LGC) models take the form:

*Y_ij_*=*I*+*St_i_*+*C_ti_*+*u_Ij_*+*u_sj_t_i_*+*u_cj_t_i_*+*e_ij_*

where *Y* is the outcome score for person, *j*, at time, *i*, *ti* is a vector of time points representing

[0, 4, 8, 12] weeks, *I* is an intercept term representing the average value at *t*=0 for all participants, *S* is a slope term representing the average linear change of *Y_ij_* over time for all

participants, *C* is a curvature term representing the quadratic change over time of *Y_ij_* for all

participants, *u_ij_* is a random intercept term representing individual participant variation in *I*, *u_sj_*

is a random slope term representing individual participant variation in *S*, *u_cj_* is a random

curvature term representing individual participant variation in *C*, and *e_ij_* is an error term.
