# Supplementary material for: Impacts of Digital Care Programs for Musculoskeletal Conditions on Depression and Work Productivity: Longitudinal Cohort Study
Source: J Med Internet Res. 2022 Jul 25;24(7):e38942. doi: 10.2196/38942 (PMC9361146; doi:10.2196/38942)
Supplement: Multimedia Appendix 2 [file jmir_v24i7e38942_app2.docx]

*Table S1. Baseline characteristics of the entire cohort.*

| Characteristic | Entire cohort  N=7785 |
| --- | --- |
| Age (years), mean (SD) |  |
|  | 51.0 (12.9) |
| Age categories, N (%): |  |
| <25 | 67 (0.9) |
| 25-40 | 1783 (22.9) |
| 40-60 | 3958 (50.8) |
| > 60 | 1977 (25.4) |
| Sex, N (%): |  |
| Female | 4274 (54.9) |
| Male | 3492 (44.9) |
| Non-binary | 19 (0.2) |
| BMI^a^, mean (SD) |  |
|  | 29.3 (6.6) |
| BMI categories^a^, N (%): |  |
| Underweight (<18.5) | 59 (0.8) |
| Normal (18.5-25) | 2124 (27.3) |
| Overweight (25-30) | 2629 (33.8) |
| Obese (30-40) | 2384 (30.6) |
| Obese grade III (>40) | 569 (7.3) |
| Conditions addressed, N (%): |  |
| Spine | 3901 (50.1) |
| Lower limb | 2157 (27.7) |
| Upper limb | 1727 (22.2) |
| Pain duration^b^, N (%): |  |
| Acute (<12 weeks) | 1923 (24.8) |
| Chronic (>12 weeks) | 5833 (75.2) |
| Employment status, N (%): |  |
| Employed (part-time or full-time) | 6616 (85.0) |
| Unemployed | 1169 (15.0) |
| Outcome measures, mean (SD) |  |
| Pain Level | 4.80 (2.0) |
| Analgesics, N (%) | 2537 (32.6) |
| Surgery Intent | 10.96 (20.41) |
| FABQ-PA^c^ | 10.62 (5.98) |
| GAD-7 | 3.09 (4.30) |
| PHQ-9 | 2.65 (4.13) |
| WPAI Overall^d^ | 17.52 (22.54) |
| WPAI Work^d^ | 16.37 (21.06) |
| WPAI Time^d^ | 2.86 (12.84) |
| WPAI Activity | 28.35 (25.15) |
| ***Missing values****:a – 20; b – 29; c -10; d N= 6030*  ***Note:*** *Significant p-values are presented in bold.* | |
