# Supplementary material for: Impacts of Digital Care Programs for Musculoskeletal Conditions on Depression and Work Productivity: Longitudinal Cohort Study
Source: J Med Internet Res. 2022 Jul 25;24(7):e38942. doi: 10.2196/38942 (PMC9361146; doi:10.2196/38942)
Supplement: Multimedia Appendix 3 [file jmir_v24i7e38942_app3.docx]

*Table S2. Unconditional Latent Growth Curve analysis: intent-to-treat analysis.*

|  |  | Trajectories parameters | | | | | | | | | Model fitness | | | | |
| --- | --- | --- | --- | --- | --- | --- | --- | --- | --- | --- | --- | --- | --- | --- | --- |
|  |  | Intercept | 95% - CI | *P* | Slope | 95% - CI | *P* | Curve | 95% - CI | *P* | Chi-sq (df) | *P* | RMSEA | CFI | SRMR |
| PHQ-9 | Cluster 1 | 0.81 | 0.77; 0.86 | **< .001** | 0.05 | 0.01; 0.09 | **.01** | 0.0 | -0.01; 0.00 | **< .001** | 191.11 (45) | < .001 | **0.035** | **0.94** | **0.026** |
|  | Cluster 2 | 6.58 | 6.44; 6.71 | **< .001** | -0.68 | -0.85; -0.52 | **< .001** | 0.03 | 0.02; 0.05 | **< .001** |  |  |  |  |  |
|  | Cluster 3 | 13.98 | 13.46; 14.50 | **< .001** | -1.52 | -1.94; -1.09 | **< .001** | 0.10 | 0.06; 0.13 | **< .001** |  |  |  |  |  |
| GAD-7 | Cluster 1 | 1.44 | 1.35; 1.53 | **< .001** | -0.04 | -0.08; 0.01 | .10 | 0.0 | 0.00; 0.00 | .97 | 111.26 (45) | < .001 | **0.024** | **0.98** | **0.013** |
|  | Cluster 2 | 5.44 | 5.07; 5.80 | **< .001** | -0.43 | -0.60; -0.27 | **< .001** | 0.02 | 0.02; 0.03 | **< .001** |  |  |  |  |  |
|  | Cluster 3 | 10.73 | 9.99; 11.47 | **< .001** | -0.75 | -1.12; -0.38 | **< .001** | 0.05 | 0.01; 0.08 | **0.010** |  |  |  |  |  |
| FABQ-PA | Cluster 1 | 10.40 | 10.18; 10.62 | **< .001** | -0.36 | -0.46; -0.26 | **< .001** | 0.01 | 0.01; 0.02 | **< .001** | 53.3 (45) | **.19** | **0.008** | **1** | **0.009** |
|  | Cluster 2 | 11.49 | 10.98; 12.01 | **< .001** | -0.24 | -0.47; -0.01 | **0.040** | 0.0 | -0.01; 0.02 | .63 |  |  |  |  |  |
|  | Cluster 3 | 12.67 | 11.92; 13.42 | **< .001** | 0.09 | -0.28; 0.45 | .64 | -0.01 | -0.04; 0.02 | .42 |  |  |  |  |  |
| WPAI Overall | Cluster 1 | 13.90 | 13.11; 14.70 | **< .001** | -1.17 | -1.52; -0.82 | **< .001** | 0.05 | 0.02; 0.07 | **< .001** | 78.37 (45) | .002 | **0.017** | **0.98** | **0.025** |
|  | Cluster 2 | 21.87 | 19.39; 24.35 | **< .001** | -2.47 | -3.51; -1.43 | **< .001** | 0.13 | 0.06; 0.20 | **< .001** |  |  |  |  |  |
|  | Cluster 3 | 39.68 | 34.86; 44.50 | **< .001** | -2.08 | -4.06; -0.11 | **.04** | 0.09 | -0.05; 0.22 | .20 |  |  |  |  |  |
| WPAI Overall^a^ | Cluster 1 | 27.26 | 26.10; 28.41 | **< .001** | -3.30 | -3.39; -2.68 | **< .001** | 0.16 | 0.11; 0.20 | **< .001** | 82.6 (45) | < .001 | **0.027** | **0.96** | **0.026** |
|  | Cluster 2 | 31.00 | 28.26; 33.74 | **< .001** | -4.17 | -5.53; -2.81 | **< .001** | 0.21 | 0.12; 0.31 | **< .001** |  |  |  |  |  |
|  | Cluster 3 | 45.43 | 40.62; 50.24 | **< .001** | -3.41 | -5.51; -1.31 | **< .001** | 0.14 | 0.00; 0.29 | .05 |  |  |  |  |  |
| WPAI Activity | Cluster 1 | 22.57 | 21.70; 23.40 | **< .001** | -1.99 | -2.34; -1.65 | **< .001** | 0.1 | 0.07; 0.12 | **< .001** | 107.2 (45) | < .001 | **0.023** | **0.98** | **0.019** |
|  | Cluster 2 | 30.99 | 28.74; 33.23 | **< .001** | -2.95 | -3.94; -1.96 | **< .001** | 0.15 | 0.09; 0.22 | **< .001** |  |  |  |  |  |
|  | Cluster 3 | 46.35 | 42.88; 49.82 | **< .001** | -1.19 | -2.86; 0.49 | .17 | 0.06 | -0.06; 0.19 | .31 |  |  |  |  |  |
| WPAI Activity^a^ | Cluster 1 | 32.04 | 31.14; 32.95 | **< .001** | -3.53 | -3.95; -3.11 | **< .001** | 0.19 | 0.16; 0.22 | **< .001** | 144.7 (45) | < .001 | **0.033** | **0.96** | **0.025** |
|  | Cluster 2 | 36.28 | 34.08; 38.48 | **< .001** | -4.01 | -5.04; -2.99 | **< .001** | 0.22 | 0.15; 0.30 | **< .001** |  |  |  |  |  |
|  | Cluster 3 | 49.50 | 46.16; 52.83 | **< .001** | -1.62 | -3.39; 0.16 | .07 | 0.09 | -0.05; 0.22 | .20 |  |  |  |  |  |
| WPAI Work | Cluster 1 | 13.04 | 12.3; 13.79 | **< .001** | -1.13 | -1.46; -0.80 | **< .001** | 0.05 | 0.03; 0.07 | **< .001** | 75.09 (45) | .003 | **0.016** | **0.99** | **0.024** |
|  | Cluster 2 | 20.78 | 18.40; 23.16 | **< .001** | -2.29 | -3.26; -1.31 | **< .001** | 0.11 | 0.05; 0.18 | **< .001** |  |  |  |  |  |
|  | Cluster 3 | 37.13 | 32.59; 41.67 | **< .001** | -1.92 | -3.81; -0.02 | **.05** | 0.07 | -0.06; 0.20 | .32 |  |  |  |  |  |
| WPAI Work^a^ | Cluster 1 | 26.06 | 24.98; 27.13 | **< .001** | -3.17 | -3.78; -2.56 | **< .001** | 0.16 | 0.11; 0.20 | **< .001** | 72.59 (45) | .006 | **0.023** | **0.97** | **0.025** |
|  | Cluster 2 | 29.58 | 26.93; 32.24 | **< .001** | -3.92 | -5.2; -2.64 | **< .001** | 0.19 | 0.10; 0.28 | **< .001** |  |  |  |  |  |
|  | Cluster 3 | 42.97 | 38.44; 47.51 | **< .001** | -3.31 | -5.37; -1.25 | **< .001** | 0.12 | -0.02; 0.27 | .09 |  |  |  |  |  |
| WPAI Time | Cluster 1 | 2.02 | 1.58; 2.46 | **< .001** | -0.04 | -0.25; 0.18 | .72 | 0.0 | -0.02; 0.01 | .62 | 75.95 (45) | .003 | **0.016** | **0.98** | **0.04** |
|  | Cluster 2 | 3.67 | 2.01; 5.34 | **< .001** | -0.68 | -1.22; -0.14 | **.01** | 0.05 | 0.01; 0.09 | **.02** |  |  |  |  |  |
|  | Cluster 3 | 13.45 | 8.94; 17.97 | **< .001** | -1.65 | -2.80; -0.50 | **< .001** | 0.1 | 0.03; 0.16 | **< .001** |  |  |  |  |  |
| WPAI Time^a^ | Cluster 1 | 27.02 | 22.52; 31.52 | **< .001** | -4.46 | -6.48; -2.44 | **< .001** | 0.17 | 0.03; 0.32 | **.02** | 138.92 (45) | < .001 | 0.096 | 0.8 | **0.076** |
|  | Cluster 2 | 25.65 | 17.24; 34.06 | **< .001** | -5.45 | -8.39; -2.50 | **< .001** | 0.31 | 0.09; 0.54 | **.01** |  |  |  |  |  |
|  | Cluster 3 | 46.10 | 35.50; 56.70 | **< .001** | -4.77 | -10.87; 1.34 | .13 | 0.24 | -0.21; 0.68 | .30 |  |  |  |  |  |

*^a^ filtered>0 score at baseline. Significant p-values are presented in bold.* *Analyzed surveys at baseline N=7785, at 4 weeks N=3865, at 8 weeks N=2766 and at 12 weeks N=2085. Trajectories were calculated through intercept (i.e., initial estimated value at baseline), slope (i.e. linear outcome change per week) and curve (i.e possible leveling effect towards the end of the program) for each variable. If a significant chi-square is found for a model, then CFI values > .9, or RMSEA values < .08, or SRMR values <.05 signify models with acceptable fit (presented in bold).[54]*
