# Supplementary material for: Impacts of Digital Care Programs for Musculoskeletal Conditions on Depression and Work Productivity: Longitudinal Cohort Study
Source: J Med Internet Res. 2022 Jul 25;24(7):e38942. doi: 10.2196/38942 (PMC9361146; doi:10.2196/38942)
Supplement: Multimedia Appendix 4 [file jmir_v24i7e38942_app4.docx]

Table S3. Conditional growth-mixture modeling analysis: intent-to-treat analysis.

|  |  | Female | | | | | | Age | | | | | | BMI | | | | | |
| --- | --- | --- | --- | --- | --- | --- | --- | --- | --- | --- | --- | --- | --- | --- | --- | --- | --- | --- | --- |
|  |  | Intercept | | Slope | | Curve | | Intercept | | Slope | | Curve | | Intercept | | Slope | | Curve | |
| PHQ-9 | Cluster 1 | **0.11** | **< .001** | **-0.01** | **< .001** | **0.02** | **< .001** | 0.01 | .53 | 0.00 | .11 | **0.01** | **.007** | 0.00 | .89 | 0.00 | .35 | **0.00** | **.02** |
|  | Cluster 2 | -0.03 | .77 | **-0.01** | **.02** | **0.02** | **< .001** | -0.02 | .86 | 0.01 | .62 | 0.00 | .97 | 0.00 | .55 | 0.00 | .69 | 0.00 | .79 |
|  | Cluster 3 | -0.29 | .38 | -0.01 | .19 | 0.03 | 0.106 | 0.19 | .39 | **0.02** | **.04** | -0.01 | .47 | -0.02 | .33 | 0.00 | .07 | 0.00 | .46 |
| GAD-7 | Cluster 1 | **0.72** | **< .001** | **-0.03** | **< .001** | 0.01 | 0.104 | -0.03 | .25 | **0.00** | **.009** | 0.00 | .13 | 0.00 | .43 | 0.00 | .14 | 0.00 | .09 |
|  | Cluster 2 | **0.87** | **< .001** | **-0.06** | **< .001** | **-0.05** | **0.01** | -0.04 | .70 | 0.00 | .37 | 0.00 | .87 | 0.00 | .59 | 0.00 | .55 | 0.00 | .95 |
|  | Cluster 3 | 0.3 | .52 | **-0.06** | **< .001** | -0.02 | 0.489 | 0.02 | .94 | **0.02** | **.02** | -0.02 | .19 | 0.00 | .89 | 0.00 | .09 | 0.00 | .35 |
| FABQ | Cluster 1 | -0.22 | .15 | **0.01** | **.03** | 0.01 | 0.62 | 0.03 | .54 | **0.00** | **.045** | **0.01** | **.02** | -0.01 | .26 | **0.00** | **.009** | 0.00 | .05 |
|  | Cluster 2 | 0.06 | .85 | 0.02 | .06 | 0.05 | 0.059 | -0.24 | .05 | 0.00 | .65 | 0.00 | .81 | 0.02 | .09 | 0.00 | .40 | 0.00 | .70 |
|  | Cluster 3 | -0.08 | .88 | **0.04** | **.04** | 0.03 | 0.3 | **-0.5** | **.02** | 0.01 | .28 | 0.00 | .94 | 0.04 | .05 | 0.00 | .77 | 0.00 | .96 |
| WPAI - Overall | Cluster 1 | **1.67** | **.004** | **-0.1** | **< .001** | **0.36** | **< .001** | -0.26 | .21 | 0.01 | .15 | -0.03 | .07 | 0.03 | .11 | 0.00 | .63 | 0.00 | .29 |
|  | Cluster 2 | 1.64 | .31 | -0.07 | .30 | 0.09 | 0.412 | 0.53 | .38 | 0.00 | .96 | -0.04 | .41 | -0.04 | .47 | 0.00 | .67 | 0.00 | .49 |
|  | Cluster 3 | -1.23 | .68 | -0.01 | .95 | 0.09 | 0.64 | 0.81 | .45 | 0.08 | .07 | -0.08 | .27 | -0.04 | .65 | 0.00 | .22 | 0.01 | .31 |
| WPAI – Overall^a^ | Cluster 1 | 1.58 | .05 | -0.04 | .24 | **0.43** | **< .001** | -0.59 | .08 | 0.00 | .81 | **-0.06** | **.02** | **0.06** | **.046** | 0.00 | .38 | 0.00 | .25 |
|  | Cluster 2 | 0.91 | .61 | -0.06 | .46 | **0.25** | **0.026** | 0.42 | .58 | -0.03 | .32 | -0.07 | .17 | -0.01 | .85 | 0.00 | .21 | 0.01 | .17 |
|  | Cluster 3 | 0.32 | .91 | 0.02 | .86 | 0.04 | 0.843 | 0.58 | .63 | 0.07 | .20 | -0.07 | .40 | -0.05 | .62 | -0.01 | .25 | 0.01 | .41 |
| WPAI - Activity | Cluster 1 | **5.23** | **< .001** | **0.1** | **< .001** | **0.48** | **< .001** | **-0.66** | **.001** | 0.01 | .46 | **-0.05** | **.005** | **0.04** | **.01** | 0.00 | .91 | **0.00** | **.03** |
|  | Cluster 2 | 2.91 | .05 | **0.27** | **< .001** | **0.47** | **< .001** | 0.63 | .25 | 0.00 | .87 | -0.02 | .60 | -0.04 | .39 | 0.00 | .91 | 0.00 | .84 |
|  | Cluster 3 | **4.83** | **.03** | **0.36** | **< .001** | **0.36** | **0.008** | **-1.85** | **.05** | 0.06 | .06 | 0.08 | .23 | 0.15 | .06 | 0.00 | .12 | -0.01 | .13 |
| WPAI – Activity^a^ | Cluster 1 | **3.63** | **< .001** | **0.24** | **< .001** | **0.04** | **< .001** | -0.28 | .26 | 0.01 | .37 | **-0.05** | **.01** | 0.01 | .51 | 0.00 | .82 | **0.00** | **.045** |
|  | Cluster 2 | 2.33 | 0.112 | **0.24** | **< .001** | **0.44** | **< .001** | 0.98 | .08 | 0.00 | .99 | -0.03 | .46 | -0.07 | .42 | 0.00 | .92 | 0.00 | .74 |
|  | Cluster 3 | **4.38** | **0.041** | **0.29** | **< .001** | **0.31** | **.02** | -1.8 | .07 | 0.06 | .05 | 0.09 | .18 | 0.15 | .08 | 0.00 | .14 | -0.01 | .11 |
| WPAI - Work | Cluster 1 | **1.56** | **0.004** | **-0.1** | **< .001** | **0.3** | **< .001** | -0.24 | .21 | 0.01 | .17 | -0.03 | .10 | 0.02 | .13 | 0.00 | .81 | 0.00 | .30 |
|  | Cluster 2 | 0.37 | 0.807 | -0.07 | .30 | 0.11 | .27 | 0.79 | .15 | 0.00 | .97 | -0.05 | .26 | -0.05 | .26 | 0.00 | .62 | 0.00 | .32 |
|  | Cluster 3 | -1.97 | 0.484 | -0.07 | .57 | 0.1 | .60 | 0.67 | .51 | 0.08 | .08 | -0.08 | .27 | -0.02 | .84 | 0.00 | .33 | 0.00 | .39 |
| WPAI – Work^a^ | Cluster 1 | **1.72** | **0.023** | -0.05 | .17 | **0.36** | **< .001** | **-0.63** | **.049** | -0.01 | .66 | -0.04 | .07 | **0.06** | **.04** | 0.00 | .26 | 0.00 | .37 |
|  | Cluster 2 | -0.24 | 0.888 | -0.07 | .34 | **0.26** | **.01** | 0.52 | .46 | -0.03 | .30 | -0.08 | .09 | -0.01 | .81 | 0.00 | .20 | 0.01 | .14 |
|  | Cluster 3 | -1.23 | 0.662 | -0.05 | .63 | 0.07 | .71 | 0.68 | .55 | 0.07 | .17 | -0.08 | .31 | -0.05 | .63 | -0.01 | .26 | 0.01 | .39 |
| WPAI - Time Missed | Cluster 1 | -0.04 | 0.883 | 0.01 | .62 | **0.12** | **< .001** | -0.02 | .83 | 0.01 | .24 | -0.01 | .41 | 0.01 | .39 | 0.00 | .26 | 0.00 | .89 |
|  | Cluster 2 | 0.85 | 0.42 | -0.02 | .73 | -0.04 | .51 | -0.06 | .85 | 0.01 | .57 | 0.03 | .07 | 0,00 | .96 | 0.00 | .63 | 0.00 | .08 |
|  | Cluster 3 | -3.97 | 0.12 | 0.16 | .10 | 0.13 | .41 | 0.96 | .13 | -0.01 | .65 | 0.01 | .89 | -0.07 | .10 | 0.00 | .88 | 0.00 | .55 |
| WPAI - Time Missed^a^ | Cluster 1 | **-8.17** | **0.004** | 0.09 | .38 | **0.49** | **.008** | 0.71 | .48 | -0.04 | .23 | -0.08 | .16 | 0.00 | .97 | 0.00 | .31 | 0.00 | .24 |
|  | Cluster 2 | -5.38 | 0.281 | 0.06 | .77 | -0.19 | .37 | 0.39 | .81 | -0.01 | .87 | **0.19** | **.03** | 0.00 | .99 | 0.00 | .95 | **-0.01** | **.03** |
|  | Cluster 3 | **-14.98** | **0.021** | 0.25 | .31 | -0.01 | .98 | 1.6 | .41 | -0.10 | .45 | -0.02 | .78 | -0.06 | .70 | 0.01 | .50 | 0.00 | .94 |

*^a^ filtered>0 score at baseline. Significant p-values are presented in bold.*
