# Supplementary material for: Addendum: Impacts of Digital Care Programs for Musculoskeletal Conditions on Depression and Work Productivity: Longitudinal Cohort Study
Source: J Med Internet Res. 2026 Aug 27;28:e97493. doi: 10.2196/97493 (PMC13563029; doi:10.2196/97493)
Supplement: Supplementary file 1 [file jmir_v28i1e97493_app1.docx]

**Table 1.** Overview of the exercise component framework, with illustrative exercise categories, progression principles, and dosage characteristics.

| **Main Goals** | **Description** |
| --- | --- |
| **Initial Phase**  · Reduce symptom burden (eg. pain, discomfort)  · Encourage gradual reintroduction of previously avoided movements  · Improve joint mobility and movement capacity  · Initiate low-load strengthening and motor control exercises | Typically, 1-2 sets per exercise, including:  · Articular mobility exercises (e.g. cervical retraction, trunk rotation)  · Strengthening exercises (e.g. supine heel slides, isometric handgrip)  · Active and passive stretching exercises (e.g. supine knee-to-chest, wrist flexors stretch)  · Condition-specific techniques when appropriate (e.g., median nerve glide in cases of suspected median neuropathy) |
| **Intermediate Phase**  · Further improve mobility and flexibility  · Progress muscular strength and endurance  · Increase tolerance to movement and activity-related symptoms  · Gradual reintroduction of functional and daily-life activities | Exercise programs were progressed based on patient response, aiming for at least 2-3 sets per exercise. Adaptations included increasing range of motion, load, or exercise dosage (sets, repetitions, session duration):  · Articular mobility exercises (e.g. pelvic tilt, standing lumbar rotation)  · Strengthening exercises (e.g. isometric wall squats, incline push ups)  · Active and passive stretching exercises (e.g. standing hip flexor stretch) |
| **Late Phase**  · Restore functional capacity  · Return to usual daily and occupational activities  · Promote long-term self-management and independence | Further increase exercise dosage (sets, repetitions, session duration) and load, increase task complexity (e.g., coordination demands):  · Articular mobility exercises (e.g. single leg knee to chest)  · Strengthening exercises (e.g. overhead press, push-ups with reduced incline)  · Active and passive stretching exercises (e.g., kneeling wrist extension) |

***Note:*** *Exercise prescription and progression was individualized based on baseline clinical assessment, ongoing feedback from the patient, performance metrics captured by the motion tracking technology during sessions, and symptom evolution (pain, fatigue, functional capacity).* *Typical Dosage (illustrative):*

*·	Recommended frequency: 3 sessions per week*

*·	Treatment duration: 8-12 weeks*

*·	Repetition ranges commonly varied (e.g., 8–15 reps depending on goal)*

*·	Programs adjusted dynamically based on tolerance and progression.*
